# Editorial: Integrative multi-omics for diagnosis, treatments, and drug discovery of aging-related neuronal diseases

**DOI:** 10.3389/fnagi.2022.1047076

**Published:** 2022-10-18

**Authors:** Chaofan Shan, Xiaoyan Liu, Yi Liu, Hantao Zhang, Junlin Liu, Yinglu Guo, Xun Gong, Min Tang

**Affiliations:** ^1^School of Life Sciences, Jiangsu University, Zhenjiang, China; ^2^Institute of Animal Science, Jiangsu Academy of Agricultural Sciences, Nanjing, China; ^3^Affiliated Hospital of Jiangsu University, Zhenjiang, China

**Keywords:** multi-omics, drug discovery, Alzheimer's disease, ischemic stroke, Parkinson's disease

The prevalence of aging-related neuronal diseases is increasing. However, there is still a huge unmet need for diagnosis, treatments, and drug discovery of these diseases. With the development of modern high-throughput omic measurement platforms, vast amounts of biological data have been generated which can be integrated in multi-omics studies to examine the complex molecular underpinnings of diseases, thus impacting the development of aging-related neuronal diseases' therapy.

In this editorial, we presented an account of how integrative multi-omics studies have greatly facilitated the diagnosis, treatments, and drug discovery of aging-related neuronal diseases. This editorial is based on 13 research articles and 3 regular reviews which shed light on the power of integrative multi-omics studies to improve aging-related neuronal diseases' therapy including but not limited to Alzheimer's disease (AD), ischemic stroke (IS), and Parkinson's disease (PD).

Several research articles analyze variables for prediction. In Han et al.'s cross-sectional study, chi square test, correlation analysis, and regression analysis were employed to analyze the influencing factors of cognitive impairment. Consequently, gender, age, education level, hypertension, and LDL-C were found to have significant differences in the incidence of cognitive impairment, providing a basis for the early screening and intervention in the elderly (Han et al.). Two research articles create prediction models based on variables of interest. Kang et al. developed an accurate, efficient nomogram with a model created by Cox regression method and further built a corresponding website to help clinicians improve their assessment of patient outcomes. In Li J. et al.'s study, they applied machine learning classification model light gradient boosting machine (LightGBM) to analyze presurgical variables of endovascular treatment (EVT) for acute ischemic stroke (AIS) induced by large-vessel occlusion (LVO) and construct a unique prediction model which was used to establish feasible and accurate presurgical prediction scale in identifying unfavorable outcomes of AIS after EVT.

Eight research articles capture gene signature (models) by integrating multi-omics information through different bioinformatics analysis and machine learning approaches. Zhang C. et al. first employed the limma R package to the got the significant differentially expressed genes (DGEs) in Depression. These DEGS were then put into weighted gene co-expression network analysis (WGCNA) and protein–protein interaction (PPI) analysis and at last the common gene general transcription factor IIF polypeptide 2 (GTF2F2) which may serve as a promising diagnostic biomarker and treatment target of depression was thus identified (Zhang C. et al.). Most of the bioinformatic approaches in this study were implemented in the article of Gu et al., where Stepwise regression and logistic regression analyses were employed to get hub genes and diagnostic model related to Iron Metabolism in AD. They also retrieved eight drugs targeting hub genes from the DrugBank database (Gu et al.). A similar study in IS was completed by Wang X. et al. where Boruta algorithm was used for genes' further screening. Importantly, they validated the gene signature with many methods, such as enrichment analyses through GO, KEGG, and GSEA pathways, ROC curves, and immune cell infiltration. Moreover, Li D. et al. demonstrated that gene methylation can also be utilized as signatures. In their research, least absolute shrinkage and selection operator cox regression analysis were carried out to construct a diagnostic signature related to PD (Li D. et al.). In Zhang W. et al.'s study, the molecular mechanism of Xingnao Kaiqiao Pill in the treatment of perioperative neurocognitive disorder (PND) was investigated from the perspective of network pharmacology and molecular docking technology. They constructed the network of “Xingnao Kaiqiao Pill–traditional Chinese medicine–compound–common target” by Cytoscape software. Molecular docking stimulation was used to further verify the interaction between the active components and key targets (Zhang W. et al.). Huang et al. and Chen Y. et al. both performed multi-omics integration analysis based on single cell technology. In their study, scRNA analysis, differential expression analysis, cell-cell communication analyses, and cell trajectory inference analyses were performed to identify candidate ligands or receptors, as well as the corresponding cell types. Combined with molecular docking, Huang et al. found that Quercetin targets VCAM1 to prevent diabetic cerebrovascular endothelial cell injury (Huang et al.), while Chen Y. et al. further identified differentially expressed transcription factors in AD associated with exercise using a modified SCENIC method. Chen Y. et al. finally constructed a network of exercise-regulating TFs in monocytes, revealing the mechanism by which exercise regulated monocytes to confer therapeutic benefits against AD and its complications. Furthermore, through target gene's knockdown and bioassays, Xia et al. found that GDF15 effectively alleviated neuronal ferroptosis post Spinal cord injury (SCI) *via* the p62-Keap1-Nrf2 signaling pathway and promoted locomotor recovery of SCI mice, which is suggested as a potential target on SCI pathogenesis and treatment.

Notably, two research articles focus on the development of computational approaches which can integrate multi-omics information. Based on topological features extracted from a protein-protein interaction (PPI) network and functional features extracted by integrating subcellular localization and homologous information of proteins, Zhu et al. exploited a novel iterative method called linear neighborhood similarity-based protein multifeatures fusion (LNSPF) to predict potential key proteins. The gene expression data downloaded from the benchmark database are used for further optimization through linear neighborhood similarity (Zhu et al.). To find biologically important imaging genetic relations more powerfully, Wang et al. imposed the GraphNet regularization penalty on the existing model and presented an improved fusion paired group lasso structured sparse canonical correlation analysis algorithm (FGLGNSCCA). Experiment results shown that the new FGLGNSCCA model proposed in their manuscript is superior or equivalent to traditional methods. With FGLGNSCCA algorithm, more AD-related biomarkers can been found (Wang et al.).

Finally, three reviews give a comprehensive understanding of the progress in the field of aging-related neuronal diseases from different aspects. Chen W. et al.'s review focuses on optogenetics in neurobiology, including how to use optogenetics to control nerve cells, study neural circuits, and treat diseases by changing the state of neurons. Wang Y. et al. revealed potential protective role of polysaccharides of Neurodegenerative Diseases (NDs), highlighting the contributions of polysaccharides and the prospects of their mechanism studies for the treatment of NDs. Ji et al. concentrated on body fluids biomarkers of early neurological deterioration (END) that have shown potential to be transferred into clinical practice.

In conclusion, the research articles and reviews in this Research Topic show how integrative multi-omics are applied to better understand and treat aging-related neuronal disease. For fully utilizing data from various omics sources to gain insights into disease, multi-omics approaches are becoming more relevant every day.

## Author contributions

This editorial was designed by MT and XG and written by CS and XL. YL, HZ, and YG had revised it. All authors have made a direct and intellectual contribution to this topic and approved the article for publication.

## Funding

This work was supported by grants from Jiangsu University (19JDG039) and the National Natural Science Foundation of China (32002235).

## Conflict of interest

The authors declare that the research was conducted in the absence of any commercial or financial relationships that could be construed as a potential conflict of interest.

## Publisher's note

All claims expressed in this article are solely those of the authors and do not necessarily represent those of their affiliated organizations, or those of the publisher, the editors and the reviewers. Any product that may be evaluated in this article, or claim that may be made by its manufacturer, is not guaranteed or endorsed by the publisher.

